# Many Atolls May be Uninhabitable Within Decades Due to Climate Change

**DOI:** 10.1038/srep14546

**Published:** 2015-09-25

**Authors:** Curt D. Storlazzi, Edwin P.L. Elias, Paul Berkowitz

**Affiliations:** 1 U.S. Geological Survey, Pacific Coastal and Marine Science Center, Santa Cruz, CA 95060 USA; 2 Deltares U.S.A., Pacific Coastal and Marine Science Center, Santa Cruz, CA 95060 USA; 3 Hawaii Cooperative Studies Unit, University of Hawaii at Hilo, HI National Park, HI 96718 USA

## Abstract

Observations show global sea level is rising due to climate change, with the highest rates in the tropical Pacific Ocean where many of the world’s low-lying atolls are located. Sea-level rise is particularly critical for low-lying carbonate reef-lined atoll islands; these islands have limited land and water available for human habitation, water and food sources, and ecosystems that are vulnerable to inundation from sea-level rise. Here we demonstrate that sea-level rise will result in larger waves and higher wave-driven water levels along atoll islands’ shorelines than at present. Numerical model results reveal waves will synergistically interact with sea-level rise, causing twice as much land forecast to be flooded for a given value of sea-level rise than currently predicted by current models that do not take wave-driven water levels into account. Atolls with islands close to the shallow reef crest are more likely to be subjected to greater wave-induced run-up and flooding due to sea-level rise than those with deeper reef crests farther from the islands’ shorelines. It appears that many atoll islands will be flooded annually, salinizing the limited freshwater resources and thus likely forcing inhabitants to abandon their islands in decades, not centuries, as previously thought.

Recent observations^1^,^2^, estimates^3^,^4^, and syntheses^5^,^6^ that include rise due to thermal expansion and ice melting show potential sea-level rise by the end of the 21^st^ century of up to 2.0 m above 2000 levels. Although the rates of sea-level rise and the ultimate elevations of sea-level rise by 2100 are debated, the existing data and predictive models all suggest that eustatic sea-level will be higher by the end of the century and that it will have a profound impact on low-lying coastal areas^6^. Sea-level rise is particularly critical for low-lying carbonate reef-lined atoll islands, many of which have maximum elevations less than 4 m above present sea level^7^,^8^. Most atoll islands have limited land and water available for human habitation, water and food sources, and ecosystems that are vulnerable to inundation from sea-level rise. Because vertical reef flat accretion rates for the coral reefs upon which carbonate islands sit (1–4 mm/yr^9^) are up to an order of magnitude smaller than the rates of projected sea-level rise (8–16 mm/yr^5^,^6^), projected sea-level rise will outstrip new reef flat accretion, resulting in a net increase in water depth over atolls’ reefs.

Sea-level rise will exacerbate the impacts of storm waves on atolls’ coral reefs by reducing wave breaking at the reef crest and increasing the water depth relative to the hydrodynamic roughness over the reef flat. This, in turn, reduces wave-energy dissipation due to interactions with the seabed^10^,^11^. By reducing incident wave energy dissipation, sea-level rise will cause larger waves to impact the coastline^12^. These larger waves will increase the resulting wave-driven set-up^11^ and thus water levels at the shoreline. Storm wave-induced water levels and the resulting flooding threatens terrestrial infrastructure and can contaminate the thin freshwater lens underlying islands with saltwater^13^. Studies to-date that describe sea-level rise threats to low-lying atoll islands^8^,^14^,^15^ generally have used passive models to simulate inundation of the islands; these “bathtub” models do not incorporate the interaction between sea-level rise and waves^16^. This study builds on preliminary efforts^17^ to explore the combined effect of sea-level rise inundation and storm-induced wave-driven flooding on atoll islands within the Northwestern Hawaiian Islands that are the home to many threatened and endangered endemic species^15^ and have high-resolution topographic and bathymetric data, yet this approach is applicable to most populated atolls around the world.

## Results

**Figure 1:**
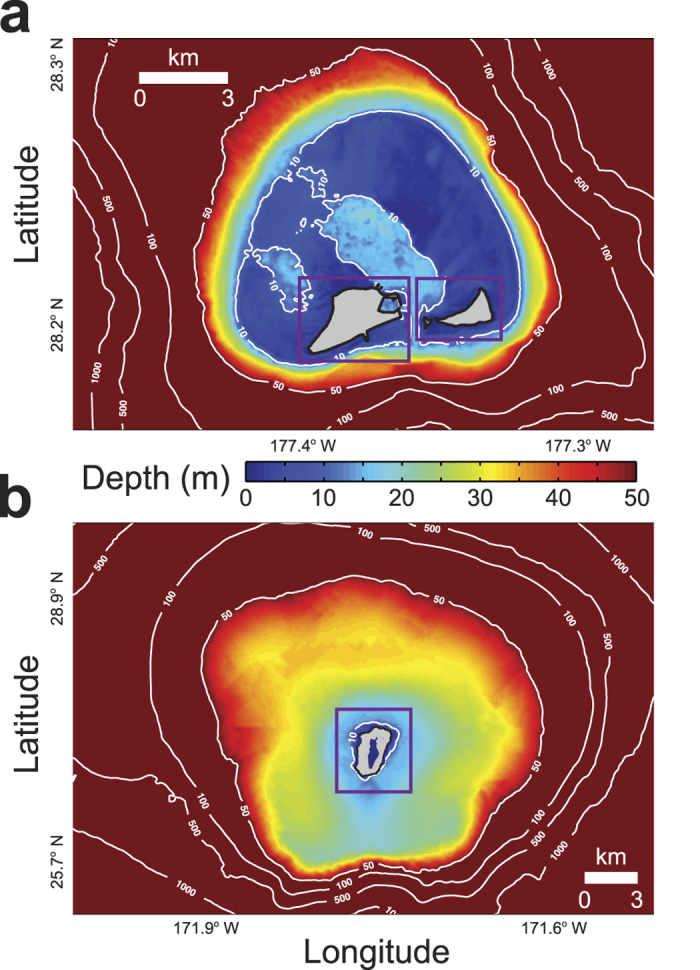
Maps showing the morphology of Midway and Laysan, Northwestern Hawaiian Islands, in the atoll-wide model grids and the smaller, finer-resolution island grids (purple boxes). (**a**) Midway. (**b**) Laysan. The 10 m, 50 m, 100 m, 500 m, and 1,000 m isobaths are labeled. Midway is characterized by islands on a shallow atoll rim and a central lagoon; Laysan, however, has a deep rim with an island in the center.

Laysan Island and Midway Atoll’s Sand, Spit, and Eastern Islands are part of the Northwestern Hawaiian Islands (Fig. 1) in the north-central Pacific Ocean. Midway Atoll (Fig. 1a), a classic atoll with islands close (<500 m) to the shallow (<5 m) carbonate rim and a deep (>10 m), central lagoon, is comprised of three islands: Sand, Spit, and Eastern Islands that have mean elevations of 3.2 m, 1.5 m, and 2.6 m, respectively. Laysan (Fig. 1b), which is characterized by a deep (>20 m) carbonate rim and an island at the center of the atoll, has mean elevation of 3.9 m. Situated in the North Pacific, these low-lying atolls are exposed to large waves. Based on 14 years of hourly hindcast data^18^ (*n* = 122,640), the average of the top 5% conditions experienced during a given winter (those experienced more than 100 hours) have wave heights, wave periods, and wave directions of 8.5 m at 15 s from 307°, and wind speeds and wind directions of 16.9 m/s from 273°, respectively. The average of top 5% of waves and winds experienced during a given summer have 3.0 m waves at 10 s from 71° and 10.4 m/s winds from 77°.

**Figure 2:**
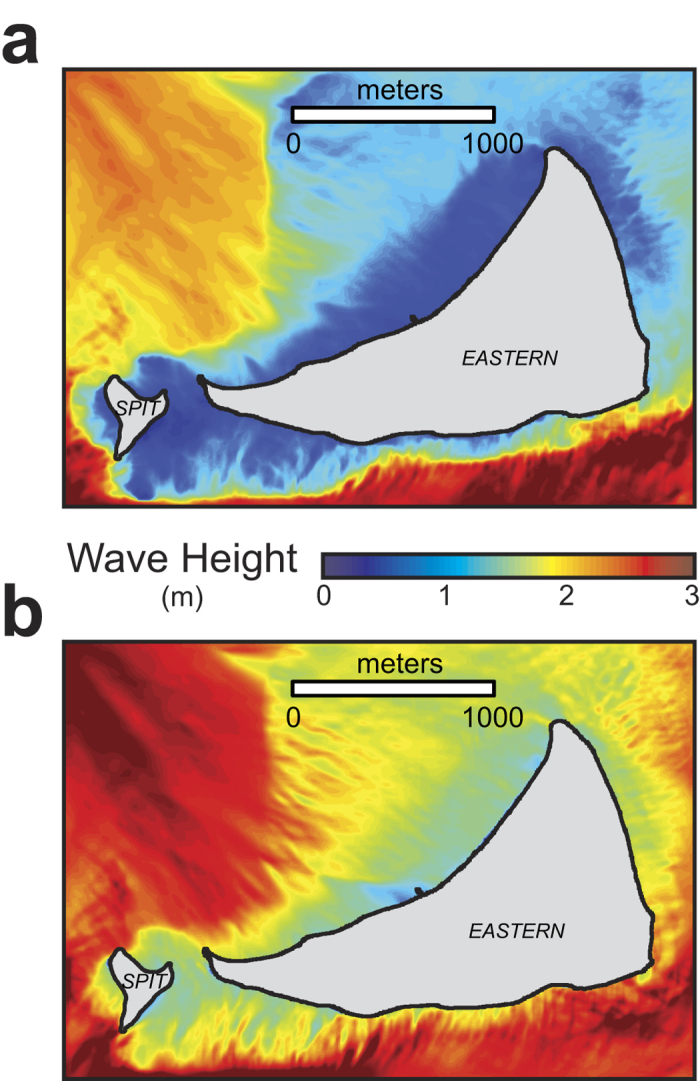
Map of modeled wave height around Eastern Island during North Pacific winter conditions. (**a**) Present sea level. (**b**) Sea level +2.0 m above present. Most reef crests and reef flats are depth-limited for waves, in that wave heights are limited to a fraction of the water depth. Thus, as water depth increases with sea-level rise over the islands’ reef flats, it allows for more deep-water wave energy to propagate onto the reef flats and more *in situ* wind-wave development on the reef flats.

**Figure 3:**
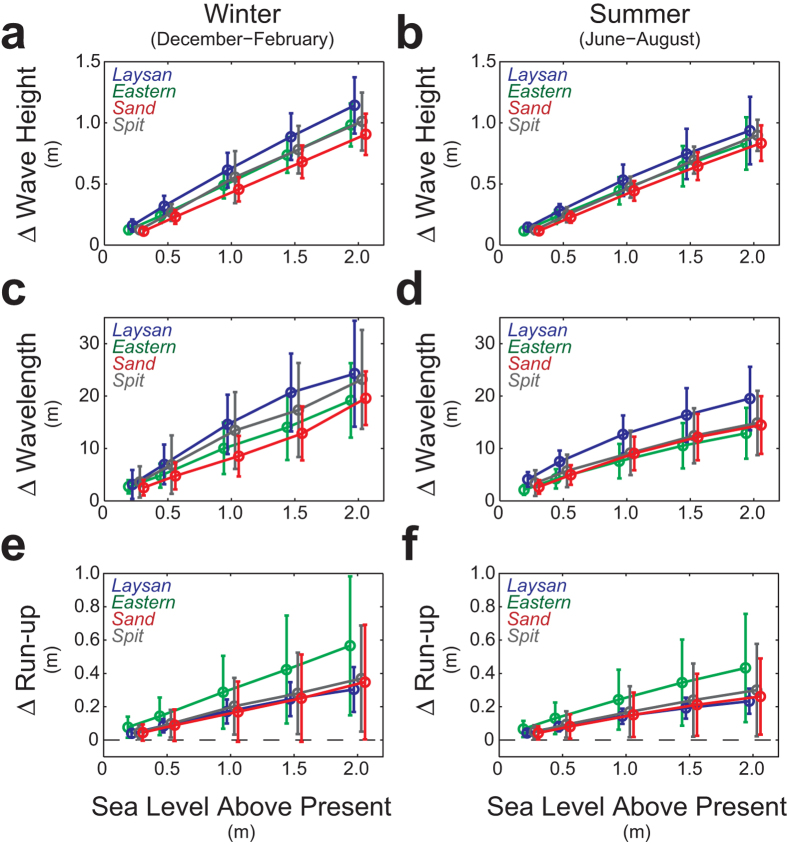
Mean changes (±1 SD) in wave parameters and the resulting wave-driven water levels around the four islands for the four sea-level-rise scenarios relative to present sea level during end-member forcing conditions. (**a**) Wave height during North Pacific winter conditions. (**b**) Wave height during North Pacific summer conditions. (**c**) Wavelength during North Pacific winter conditions. (**d**) Wavelength during North Pacific summer conditions. (**e**) Run-up during North Pacific winter conditions. (**f**) Run-up during North Pacific summer conditions. *N* = 3321, 416, 1086, and 257 for Sand, Spit, Eastern, and Laysan, respectively. Wave height and length increase with sea-level rise due to reduced wave breaking and hydrodynamic roughness relative to water depth. The increases in wave height and wavelength result in much greater run-up at higher sea levels.

Waves on most atoll reef crests and reef flats are water depth-limited, which suggests as water depth increases with sea-level rise, larger waves^17^ will propagate onto or develop via wind forcing on the reef flat or in the lagoon (Fig. 2). Wave heights along the islands’ shorelines will become larger with rising sea level. Because wavelength in shallow water is primarily a function of the ratio of wave period to water depth, wavelengths will also increase with sea-level rise (Fig. 3). Mean wave-driven run-up adjacent to the low-lying atoll islands, which is a function of wave height, wavelength, and the slope of the beachface^19^, will similarly increase with sea-level rise. Although there is large heterogeneity in the resulting wave-driven run-up around the islands^17^ due to spatial variations in waves around the islands (Fig. 2) and beachface slope, there is a general trend of higher wave-driven run-up on the sides of the islands closer to the reef crest. This is related to greater wave and resulting set-up that influences depth-limited wave heights on the reef flat because less wave energy is dissipated on the narrower reef flats.

**Figure 4:**
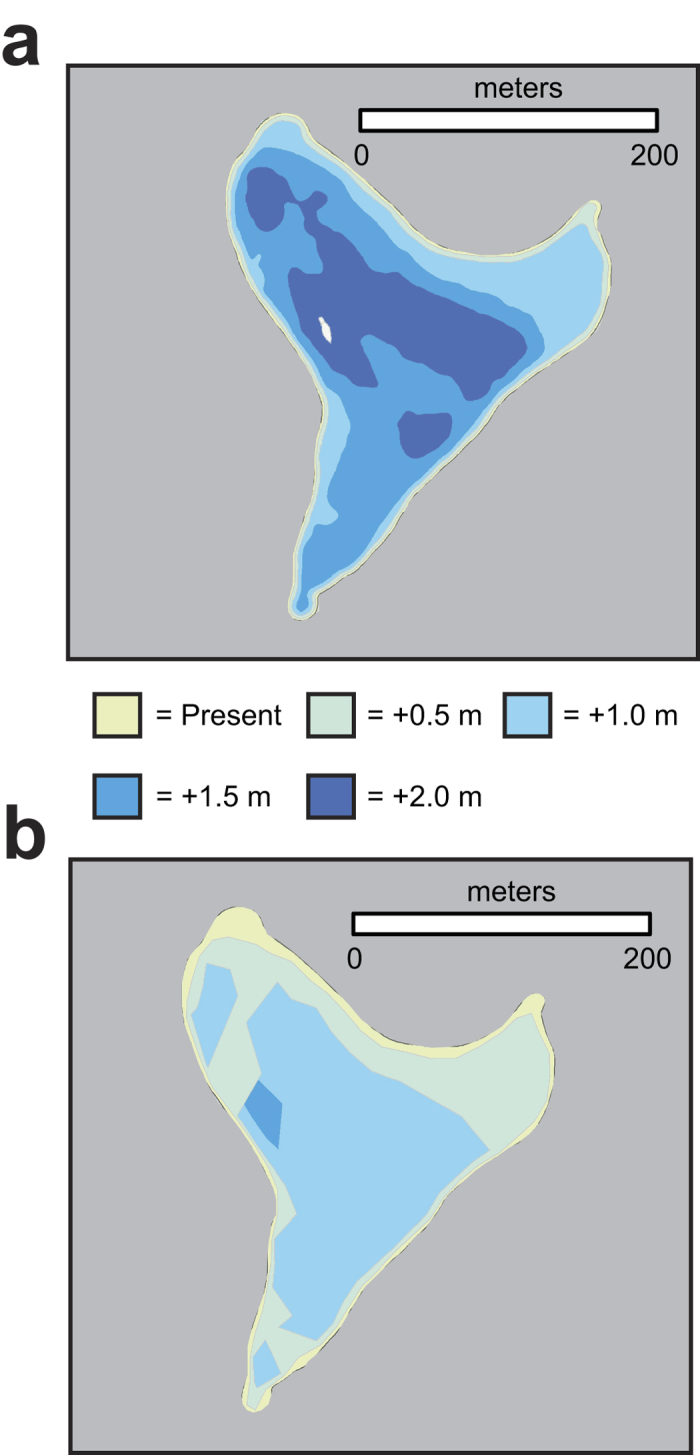
Maps of inundation or flooding limits at Spit Island for the four sea-level-rise scenarios relative to present sea level showing the differing results from the two types of models. (**a**) Passive inundation modeling. (**b**) Dynamic wave-driven flood modeling. The passive models predict less than 1% of Spit Island remain dry at with 2.0 m of sea-level rise. The dynamic models, however, predict a similar extent of impact with 1.0 m of sea-level rise and for the entire island to be flooded multiple times annually with 1.5 m of sea-level rise.

**Figure 5:**
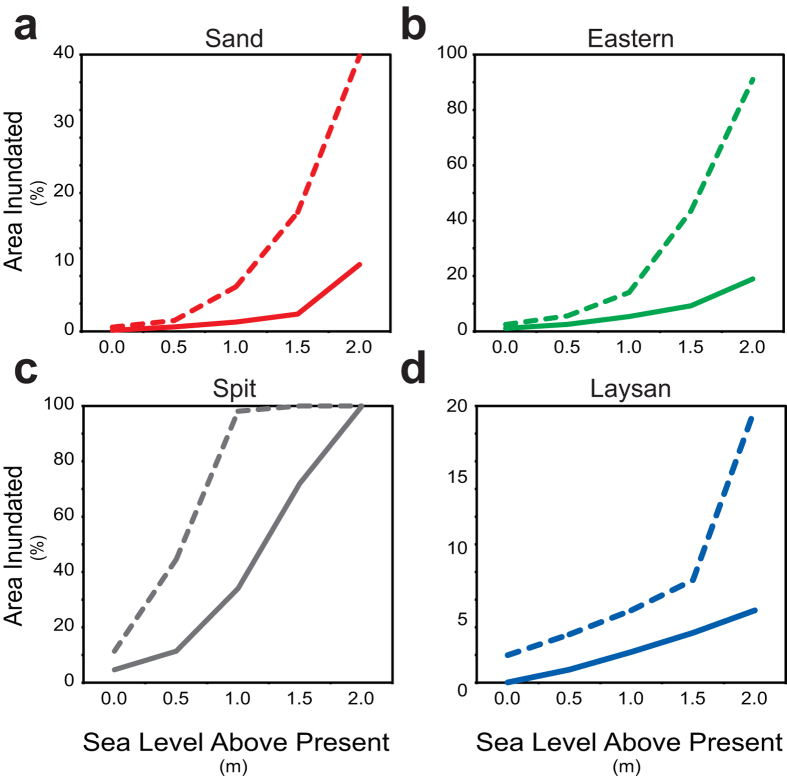
Differences in the percentage of land area on the four islands projected by passive modeling to be inundated (solid lines) versus that flooded by dynamic modeling (dashed lines) for the four sea-level rise scenarios relative to present sea level. (**a**) Sand Island. (**b**) Eastern Island. (**c**) Spit Island. (**d**) Laysan Island. The dynamic models that include wave-driven water levels forecast much greater impact for a given water level, or sooner in the future, than passive models.

Across all sea-level-rise scenarios, the extent of inundation predicted under the passive modeling approach covers less area than the flooding extent forecasted by the wave-driven water level approach, as shown in Fig. 4 for Spit Island. For Sand, Eastern, and Laysan Islands, less than 19% of the islands’ areas are forecast to be inundated with sea-level rise = 2.0 m (Fig. 5). Spit Island, which is much lower lying, becomes fully inundated when sea-level rise = 2.0 m. When passive sea-level-rise is combined with wave-driven water levels, similar patterns of impact occur at sea-level rise scenarios less than 1.0 m (Fig. 5). Projected flooding doubles from sea-level rise = 0.0 m to 0.5 m for all islands except Spit, where sea-level rise and wave-driven water levels cause the projected flooding to quadruple to more than 44% of the island’s area. The dynamic modeling approach that incorporates wave-driven effects projects Spit Island to have approximately the same area flooded at sea-level rise = 1.0 m (96.5%) as is projected to be inundated by the passive modeling when sea-level rise = 2.0 m (98.4%). On Sand, Eastern, and Laysan Island, when sea-level rise = 1.5 m and 2.0 m, the flooding patterns start to diverge considerably from passive inundation patterns, as water levels begin to exceed the existing coastal berm and extend considerable distances inland over low-lying areas. Eastern Island shows the greatest difference between the passive inundation and dynamic flood modeling when sea-level rise = 2.0 m, for the passive modeling suggests an inundation extent of 18%, whereas the dynamic modeling predicts flooding to impact to 90% of the island’s area.

## Discussion

As shown previously^17^, it is apparent that the passive “bathtub” modeling predicts less inundation than the dynamic flood modeling for these low-lying atoll islands. These differences between the passive and dynamic modeling are especially true for higher sea-level-rise scenarios (Fig. 5); these results can be viewed two ways. First, more of the atoll islands will be periodically inundated at a given future sea level when taking wave-driven processes into account than predicted by the passive models. Second, because global sea level is expected to rise for the foreseeable future, the sea-level-rise scenarios used here can be assumed to be somewhat interchangeable with time. Thus, the dynamic flood model results provided here suggest that a given percentage of the low-lying atoll islands will be annually impacted at lower values of sea-level rise, and thus sooner in the future, than predicted by the passive models.

The results from this modeling study also call attention to the role of low-lying atoll islands’ geomorphology on susceptibility to sea-level rise and wave-induced impacts. Although dynamic flood modeling predicted up to an order of magnitude greater inundation than the passive inundation methodology, Midway’s Sand, Spit, and Eastern Islands were modeled to undergo much greater impact for a given value of sea-level rise using both types of models than Laysan Island. Midway’s islands, which lie close to the shallow rim on this classic atoll, are subjected to smaller nearshore wave heights and shorter wavelengths due to depth-limited breaking of deep-water waves on the shallow reef crests of the carbonate rim. However, because predicted rates of sea-level rise will outstrip vertical reef flat accretion, depth-limited breaking on the reef crests will be significantly reduced by sea-level rise. This will result in greater wave heights and wavelengths on the atoll’s reef flats than at present, higher wave-driven water levels along the islands’ shorelines, and greater flooding. Laysan, on the other hand, which is characterized by a deep (>20 m) carbonate rim and a central island far from the atoll rim, allows larger wave heights to propagate closer to shore than at Midway. Because of this, Laysan has steeper coastal topography and a higher mean elevation than islands on a classic atoll. The more energetic nearshore wave environment and resulting steeper and higher coastal topography results in Laysan Island undergoing much less flooding for a given sea-level-rise scenario than Midway’s islands. Together, these modeling results and observations demonstrate that classic atolls with low-lying islands close to the shallow atoll rim are more susceptible to the combined effects of sea-level rise and wave-driven flooding than low-lying atoll islands farther from a deeper carbonate rim.

Models of sea-level-rise impacts to low-lying atoll islands that incorporate annual storm wave dynamics provide insight into the potential flooding that would occur multiple times during the average winter. This is in contrast to a once-in-a-decade or so flood event modeled using 10-, 20-, or 50-year storms that might allow humans^20^,^21^, freshwater^13^,^20^, vegetation^19^, and wildlife populations^15^ to recover quickly due to their infrequency relative to the speed of infrastructure repair, rate of lens re-freshening and vegetation regeneration, or wildlife population dynamics (e.g., annual breeding cycle). Rather, the use of typical storm-wave conditions predicts events that would occur a few times every year and thus become a part of the annual cycle. This would potentially influence society via impacts to infrastructure and/or freshwater supplies if the rate of re-freshening is longer than the recurrence interval of the overwash events^13^ or wildlife distributions through reducing reproductive success of species that exhibit breeding site fidelity that are vulnerable to habitat inundation^21^.

Current efforts to predict how long atolls may be inhabitable have relied solely on passive “bathtub” inundation modeling, suggesting that many atoll islands may remain suitable for human habitation for 50–150 years^8^,^14^. The results presented here, however, predict greater spatial impact, especially at higher sea-level-rise scenarios, when wave-driven flooding is taken into account. If, as discussed before, sea-level-rise is therefore interchangeable with time, then the dynamic model results that incorporate wave-driven processes provided here suggest low-lying atoll islands will be annually flooded by seawater sooner in the future than predicted by the passive models. Not only will such flooding impact terrestrial infrastructure and habitats, but more importantly, it will salinize the limited freshwater resources^13^,^20^ and thus may force inhabitants to abandon their island-nations in decades, not centuries, as previously thought^8^,^14^. Together, these results highlight the synergistic effects of annual storm waves and sea-level rise to provide an improved understanding of the planning and management necessary to protect infrastructure and natural resources on low-lying atoll islands in the face of future climate change.

## Methods

### Oceanographic and meteorologic forcing

The potential impact of sea-level rise inundation and flooding from ocean waves on Laysan Island and Midway Atoll’s Sand, Spit, and Eastern Islands were investigated using numerical wave - and water-level models driven by hindcast data and projected values of sea-level rise^17^. There are no wave buoys with long records for the study area. Accordingly, hourly hindcast wind and wave data^18^ from 1981–2004 were used to constrain the primary wave sources (North Pacific, trade wind, South Pacific, and hurricane waves) for the area. These data were analyzed for top 5% storm conditions because they represent conditions exceeded 36 h per month, and are most likely to coincide with a high tide and thus characterize maximum water level conditions experienced in a given year. Forecasts of the wind and wave climate in the study area^22^,^23^ are not in firm agreement on potential change and therefore this study does not take into account any future change in forcing. Because of the large range of forcing conditions, the data presented here are constrained to the two end-members: North Pacific winter and summer conditions that encompass the sources described above; spring and fall conditions fall between these two end-members.

### Numerical modeling

The hindcast deep-water waves and winds were used to drive simulations of waves and water levels on the atolls using Deltares’ Delft3D hydrodynamic model. Delft3D simulates water levels via the Delft3D-FLOW module, which is coupled to the SWAN wave model^24^,^25^. These models have been shown to accurately model the propagation and breaking of waves and the resulting water levels over Pacific coral reefs^12^,^26^,^27^, and specifically Midway Atoll^28^. On reefs, wave breaking occurs generally at two locations – at the reef crest and along the coastline. Because wave breaking causes gradients in water-levels, wave breaking results in elevating water levels on the reef flat, then again at the shoreline. The elevation in water over the reef flat due to wave breaking at the reef crest^12^,^27^ has been referred to positive sea-level anomalies^28^, whereas the superelevation of water along the shoreline in the beach’s surf zone^12^ is referred to as set-up. The SWAN output, such as wave height, wave period, and wave-driven water levels (positive sea-level anomalies and set-up) are coupled with the Delft3D-FLOW, providing updated water levels over which to compute the updated wave fields.

### Bathymetric and topographic data

Most of the standard model settings were used, based on the previous successful implementation at Midway Atoll^28^. Seamless nested topographic/bathymetric grids were created as boundary data for the models. Larger atoll grids covered a broad area surrounding the entire atoll to water depths of 3000 m and had coarser spatial resolutions and larger grid cell sizes (Fig. 1); finer grids encompassing the islands plus the surrounding waters had higher spatial resolutions and were embedded into the larger, coarser grids. Midway’s atoll grid had 180,851 cells at 50-m resolution, the Sand Island grid had 147,000 cells at 20-m resolution, and the Spit and Eastern Islands’ grid had 73,125 cells at 20-m resolution^29^. Laysan’s atoll grid had 175,851 cells at 100-m resolution and the Laysan Island grid had 85,914 cells at 20-m resolution^17^,^30^; all of these were referenced to mean higher water. The methodology and bathymetric grids presented here reflect the current state of the reefs without future reef accretion because no data exist for the study areas and published vertical reef flat accretion rates for reef flats exposed to open-ocean storm waves (1–4 mm/yr^10^) are very small compared with the rates of projected sea-level rise until 2100. The results from the larger, outer domains for the larger models that covered the entire atolls were used as boundary input into the finer-scale, smaller sized domains in models around the atoll islands. The models were run with open water-level boundaries defined by astronomic forcing; the water level was adjusted for the four sea-level rise scenarios by defining an A0 tide amplitude (+0.5 m, +1.0 m, +1.5 m, and +2.0 m) with 0° phase.

### Inundation and flood modeling

SWAN output wave heights and wavelengths were used as input for wave run-up computations at each grid cell in the surf zones adjacent to the islands’ sandy shorelines. Unlike preliminary efforts^17^ that utilized equations developed for sandy shorelines, an empirical methodology^19^ that utilizes breaking wave parameters at the foot of the slope was used to provide more accurate measures of wave-driven run-up for reef-lined coasts. For each grid cell, wave-induced set-up, wave-driven run-up heights, and sea-level-rise values were combined into total water elevations and then projected on the islands’ slopes along shore-normal transects to outline areas flooded. In order to map inundation and flooding on the islands, the modeled sea-level rise and wave-driven water-level surface was projected orthogonally upslope from each coastal point until land elevations exceeded combined water heights, with the highest point reached on each transect representing the high water mark. The extent of inundation and flooding was delineated by connecting high water marks from adjacent transects.

As a refinement to the above method, to model inundation and flooding, water was modeled to flow into depressions if they were adjacent to flooded transects, hydrologically connected, and at lower elevations than the flooded transects. Although the empirical methodology^19^ produces run-up elevations, it does not estimate run-up volumes; therefore, the volume of run-up that is likely to flow into adjacent topographic depressions is unknown. Thus in depicting the extent of inundation for sea-level rise scenarios, the initial inundation patterns were mapped as wave-driven flooding propagates onshore, representing a transient state before run-up volumes flow laterally into adjacent depressions. Based on the long average duration of storms (>6 hours) and high frequency of swash motions (order ~12–900 times/hour) relative to hydraulic conductivities of such material (<0.0001 m/s), it was assumed that on the time-scale of wave-driven flooding events, infiltration is approximately non-existent and thus the maximum extent of flooding was delineated as if run-up volumes were unlimited and no infiltration occurred. This allowed seawater to fill the topographic lows during storm events. For both the passive and dynamic wave-modeling approaches, inundation and flooding extents were mapped for all five sea-level rise scenarios. The passive approach depicts the amount of inundation above mean higher water due to sea-level rise only, while the dynamic approach considers the additional effect of wave-driven set-up and run-up from the average of the top 5% storm conditions in the hindcast forcing data^18^.

## Additional Information

**How to cite this article**: Storlazzi, C. D. *et al.* Many Atolls May be Uninhabitable Within Decades Due to Climate Change. *Sci. Rep.* **5**, 14546; doi: 10.1038/srep14546 (2015).
